# Exploration of *Deinococcus-Thermus* molecular diversity by novel group-specific PCR primers

**DOI:** 10.1002/mbo3.119

**Published:** 2013-08-29

**Authors:** Nicolas Theodorakopoulos, Dipankar Bachar, Richard Christen, Karine Alain, Virginie Chapon

**Affiliations:** 1 CEA, DSV, IBEB, SBVME, LIPMF-13108, Saint-Paul-lez-Durance, France; 2 CNRS, UMR 7265F-13108, Saint-Paul-lez-Durance, France; 3 Université d'Aix-MarseilleF-13108, Saint-Paul-lez-Durance, France; 4 IRSN, PRP-ENV, SERIS, L2BTF-13115, Saint Paul-lez-Durance, France; 5 Université de Nice-Sophia Antipolis, UMR 7138, Systématique, Adaptation, Evolution, Parc Valrose, BP71F-06108, Nice Cedex 02, France; 6 CNRS, UMR 7138, Systématique, Adaptation, Evolution, Parc Valrose, BP71F-06108, Nice Cedex 02, France; 7 CNRS, IUEM – UMR 6197, Laboratoire de Microbiologie des Environnements Extrêmes (LMEE)Place Nicolas Copernic, F-29280, Plouzané, France

**Keywords:** *Deinococcus-Thermus*, group-specific primers, molecular diversity

## Abstract

The deeply branching *Deinococcus-Thermus* lineage is recognized as one of the most extremophilic phylum of bacteria. In previous studies, the presence of *Deinococcus*-related bacteria in the hot arid Tunisian desert of Tataouine was demonstrated through combined molecular and culture-based approaches. Similarly, *Thermus*-related bacteria have been detected in Tunisian geothermal springs. The present work was conducted to explore the molecular diversity within the *Deinococcus-Thermus* phylum in these extreme environments. A set of specific primers was designed in silico on the basis of 16S rRNA gene sequences, validated for the specific detection of reference strains, and used for the polymerase chain reaction (PCR) amplification of metagenomic DNA retrieved from the Tataouine desert sand and Tunisian hot spring water samples. These analyses have revealed the presence of previously undescribed *Deinococcus-Thermus* bacterial sequences within these extreme environments. The primers designed in this study thus represent a powerful tool for the rapid detection of *Deinococcus-Thermus* in environmental samples and could also be applicable to clarify the biogeography of the *Deinococcus*-*Thermus* phylum.

## Introduction

The phylum *Deinococcus-Thermus* is currently divided into the orders *Deinococcales* and *Thermales*. While the order *Thermales* encompasses five genera (*Thermus*, *Meiothermus*, *Marinithermus*, *Oceanithermus,* and *Vulcanithermus*), the order *Deinococcales* is composed of three genera (*Deinococcus, Deinobacterium,* and *Truepera). Deinococcus-Thermus* is recognized as one of the most extremophilic phylum of bacteria. Cultured representatives of *Thermus* are either thermophilic or hyperthermophilic (Brock and Freeze 1969; Bjornsdottir et al. 2009; Zhang et al. 2010; Vajna et al. 2012), while *Deinococcus* strains exhibit resistance to extreme ionizing and ultraviolet radiations, desiccation, and other DNA damaging conditions (Rainey et al. 1997; Albuquerque et al. 2005; Cox and Battista 2005; Slade and Radman 2011). As these microorganisms or their cellular components are of biotechnological interest with potential applications in bioremediation or molecular biology (e.g., thermostable enzymes), much research has been focused on this particular group of prokaryotes. *Deinococcales* members have been isolated from a wide range of natural environments such as arid soils (Hirsch et al. 2004; de Groot et al. 2005; Rainey et al. 2005, 2007; Chanal et al. 2006; Callegan et al. 2008; Yuan et al. 2009), radioactive sites (Asker et al. 2011), nuclear waste contaminated sediments (Fredrickson et al. 2004), air (Yoo et al. 2010), water (Im et al. 2008; Kämpfer et al. 2008), and the human gut (Lagier et al. 2012); they have also been isolated from a paper mill (Ekman et al. 2011). *Thermales* members have been recovered from a large set of natural and artificial thermal environments (Oshima and Imahori 1974; da Costa et al. 2006; Vajna et al. 2012; Yu et al. 2012), highlighting the exceptional adaptive abilities of these bacteria. In addition, *Deinococcus-Thermus*-related sequences have been detected by molecular tools in a huge range of biotopes, where they generally represent minor taxa (Janssen 2006).

In previous molecular diversity inventories, we have demonstrated the presence of *Deinococcus* taxa in the desert sands of Tataouine, Tunisia (Chanal et al. 2006), as well as the presence of *Thermus* taxa in Tunisian geothermal springs (Sayeh et al. 2010). However, the 16S rDNA clone libraries constructed from these studies are insufficient for a thorough exploration of *Deinococcus-Thermus* diversity. Indeed, sequences belonging to these taxa represent less than 2% of the overall community detected in Tataouine, and less than 10% of the overall revealed community in the Tunisian hot springs. An alternative approach to investigate the diversity of a taxonomic group in greater detail is to use group-specific primers specifically targeting a given taxon. This has been a successful strategy for the detection of diverse groups of Bacteria and Archaea at different taxonomic levels, including *Actinobacteria* (Stach et al. 2003); *Bacteroidetes, Planctomycetes, Firmicutes, Cyanobacteria, α-, β-,* and *γ-proteobacteria* (Mühling et al. 2008); *Korarchaeota* (Auchtung et al. 2011); *Acidobacteria* (Lee and Cho 2011; Gans et al. 2012); *Pseudomonas* (Widmer et al. 1998); and *Francisella* (Duodu et al. 2012). Furthermore, a specific primer for hemi-nested polymerase chain reaction (PCR) that targets the genus *Deinococcus* has recently been described (Chaturvedi and Archana 2012).

Here, we have developed specific PCR primers that target the 16S rRNA gene sequence of the entire *Deinococcus-Thermus* phylum. Following the initial in silico design step, primers specificity was tested with a collection of reference strains. Finally, we constructed 16S rDNA clone libraries to validate the use of these primers with environmental DNA. This approach has enabled us to detect novel representatives of *Deinococcus* and *Thermus* in desert sand samples from Tataouine, as well as in Tunisian geothermal spring water.

## Material and Methods

### Bacterial strains, culture conditions, and genomic DNA purification

The bacterial strains used in this study are listed in Table 1. Biomass for the genomic DNA extraction was prepared by growing *Deinococcus* isolates in TGY medium (0.5% tryptone, 0.1% glucose, 0.3% yeast extract) at 30°C; *Escherichia coli* and *Pseudomonas aeruginosa* were cultivated in Luria-Bertani (LB) medium (1% tryptone, 0.5% yeast extract, 1% NaCl) at 37°C; *Shewanella oneidensis* was grown in LB medium at 30°C; all other isolates were grown in 0.1× Tryptic Soy Broth (DIFCO laboratories, Detroit, MI) at 30°C. Bacteria were harvested by 5 min centrifugation at 10000*g*, and DNA extractions were performed on the cell pellet with the DNeasy® Blood & Tissue kit (Qiagen, Hilden, Germany), according to manufacturer's instructions. *Magnetospirillum magneticum* genomic DNA was provided by J. B. Rioux (IBEB-LBC-CEA Cadarache, France).

**Table 1:** Bacterial strains used in this study

Bacterial strains	Description, origin, reference number	Reference or source
*α-proteobacteria*		
*Rhodobacter sphaeroïdes* 2.4.1	Type strain (ATCC 11167)	M. Sabaty, CEA Cadarache, France
*Magnetospirillum magneticum* AMB-1	Type strain (ATCC 700264)	J. B. Rioux, CEA Cadarache, France
*Afipia* sp. CYB52	Arsenic contaminated water, Bangladesh	Lab. collection
*Brevundimonas* sp. OVA 3.2	Soil, Gabon	Lab. collection
*Chelatococcus* sp. VCT108	Tataouine desert sand, Tunisia	Chanal et al. (2006)
*β-proteobacteria*		
*Variovorax* sp. Cu5	Uranium ores, France	Lab. collection
*Burkholderia* sp. VeU10	Uranium ores, France	Lab. collection
*Collimonas* sp. Ve03a1 ou VeU15	Uranium ores, France	Lab. collection
*γ-proteobacteria*		
*Escherichia coli* MG1655	Type strain (ATCC 700926)	Lab. Collection
*Pseudomonas aeruginosa* PAO1	Type strain (ATCC BAA-47™)	R. Voulhoux, CNRS, France
*Shewanella oneidensis* MR1	Type strain (ATCC BAA-1096™)	A. Verméglio, CEA Cadarache, France
*Deinococcus-Thermus*		
*Deinococcus radiodurans* R_1_	Type strain (ATCC 13939)	S. Sommer, Univ. Of Paris-Sud, France
*Deinococcus desertii* VCD115	Type strain (DSM 17065)	de Groot et al. (2005)
*Deinococcus desertii* VCD117	Sahara desert, reference strain	de Groot et al. (2005)
*Deinococcus* sp. VCT102	Tataouine desert sand, Tunisia	Chanal et al. (2006)
*Deinococcus proteolyticus* MRP	Feces of *Lama glam,* type strain (ATCC 35074)	Kobatake et al. (1973); Brooks and Murray (1981)
*Deinococcus radiopugnans* MIT 248	Irradiated haddock, type strain (ATCC 19172)	Brooks and Murray (1981)
*Deinococcus murrayi* ALT-1b	Hot springs, type strain (DSM 11303)	Ferreira et al. (1997)
*Deinococcus geothermalis* AG-3a	Hot springs, type strain (DSM 11300)	Ferreira et al. (1997)
*Deinococcus indicus* Wt/1a	Arsenic polluted water, type strain (DSM 15307)	Suresh et al. (2004)
*Deinococcus radiophilus* RBD	Irradiated Bombay duck, type strain (DSM 20551)	Lewis (1973); Brooks and Murray (1981)
*Deinococcus grandis* KS 0485	Hot springs, type strain (DSM 3963)	Oyaizu et al. (1987); Rainey et al. (1997)
*Meiothermus ruber* 21	Hot springs, type strain (DSM 1279)	Loginova et al. (1984); Nobre et al. (1996)
*Thermus aquaticus* YT-1	Hot springs, type strain (DSM 625)	Brock and Freeze (1969)
*Thermus thermophilus* HB8	Hot springs, type strain (DSM 579)	Oshima and Imahori (1974); Manaia et al. (1994)
*Firmicutes*		
*Lysinibacillus* sp. Vi07	Granitic soil, France	Lab. collection
*Paenibacillus* sp. Vi0A7b	Granitic soil, France	Lab. collection
*Bacillus* sp. ViU12	Uranium ores, France	Lab. collection
*Actinobacteria*		
*Corynebacterium* sp. Vi06	Granitic soil, France	Lab. collection
*Streptomyces* sp. Vi02	Granitic soil, France	Lab. collection
*Arthrobacter* sp. ViUA5	Uranium ores, France	Lab. collection
*Leifsonia* sp. Ve03a2	Granitic soil, France	Lab. collection
*Bacteroïdetes*		
*Pedobacter* sp. VeU6	Uranium ores, France	Lab. collection
*Chryseobacterium* sp. TchI_1_n3	Radionuclide-contaminated soil, Chernobyl	Chapon et al. (2012)
*Hymenobacter* sp. TchI_11_n5	Radionuclide-contaminated soil, Chernobyl	Lab. collection
*Sphingobacterium* sp. CYB21	Arsenic contaminated water, Bangladesh	Lab. collection

### Environmental DNA

**Figure 1:**
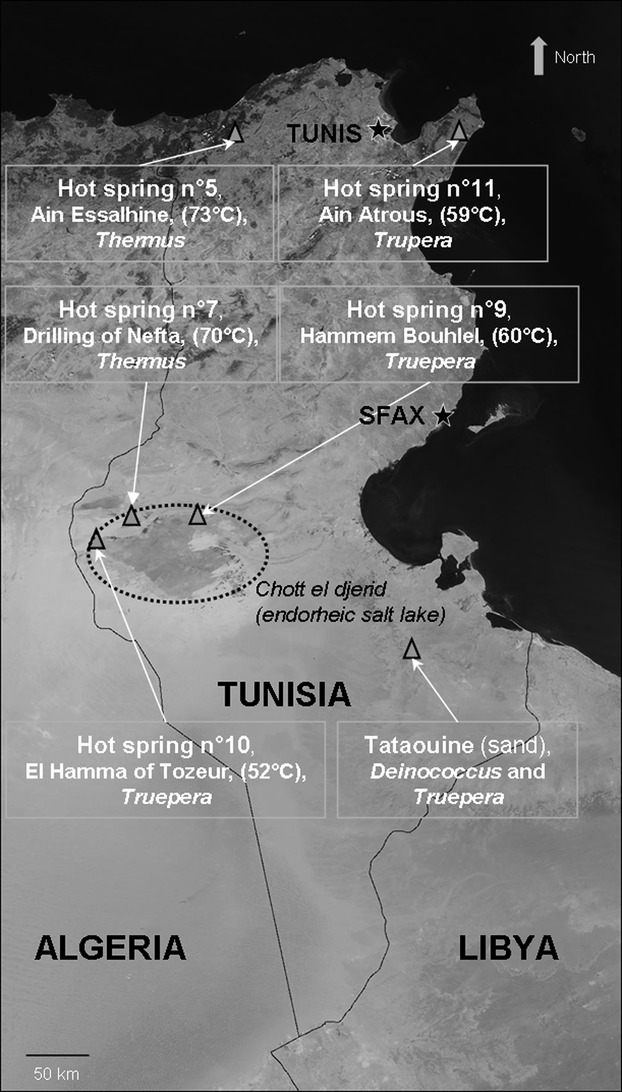
Location map of the study sites (triangles). For the hot springs, the water temperature is indicated in brackets. Genera belonging to the *Deinococcus-Thermus* phylum retrieved in each site are also indicated.

Sample collection and environmental DNA extraction for the sample from the Tataouine desert (located ∼100 km west from the Sahara border; see Fig. 1) are reported in Chanal et al. (2006). *Deinococcus* have been detected therein by a combination of molecular and cultural methods. The present study was performed with purified DNA stored at −20°C.

Five water samples from Tunisian geothermal springs, described in Sayeh et al. (2010), were examined in this study: Ain Essalhine (spring 5), Nefta (spring 7), Hammem Bouhlel (spring 9), El Hamma of Tozeur (spring 10), and Ain Atrous (spring 11) (see Fig. 1). Springs 5, 9, and 11 were chosen as control samples as *Thermus* sequences have been previously detected therein. Springs 7 and 10 were also examined to assess whether the use of specific primers is more sensitive than the cloning/sequencing approach, as no *Deinococcus/Thermus* were detected previously. Water samples (either 10 mL or 50 mL) were filtered through 0.2-μm pore size filters, which were then stored at −80°C. Environmental DNA was extracted directly from these filters in the present study, using the UltraClean Soil DNA Isolation Kit (MoBio, Solana, CA).

### In silico design of *Deinococcus-Thermus* specific primers

All *Deinococcus-Thermus* sequences were extracted from the SILVA 111 reference sequences and aligned using the Muscle program. From this, a 90% sequence consensus was computed, and encoded using the IUPAC notation (without taking into account indels for consensus computing). A 15 nucleotide (nt) sliding window was used to extract each subsequence containing less than three degeneracies. The overlapping extracted 15 nt oligomers were then recombined into longer domains. Each possible primer with a length of 20–30 nts containing less than three degeneracies was then extracted from the domains.

A specific program, written in C, was developed to test which sequences would be recognized in the SILVA database, allowing up to three mismatches. Primers were then selected having a high specificity and a wide coverage for sequences of the *Deinococcus-Thermus* clade. A set of Python programs was used to improve their coverage while remaining specific. At the end of this process, the Deino-f-326-350/Deino-r-758-785 primers displayed good coverage and specificity, and we selected them for in vivo validation.

### PCR amplification and construction of 16S rRNA gene libraries

For the in vitro validation of primers that specifically target *Deinococcus-Thermus*, an initial set of experiments was performed using phylogenetically diverse bacteria (Table 1). Genomic DNA from pure cultures was used as a template for PCR amplification with the primers Deino-f-326-350 (5′-CGGGAGGCAGCAGTTAGGAATCTTC-3′) and Deino-r-758-785 (5′-GTTTAGGGYGTGGACTACCCGGGTATCT-3′). Each amplification reaction mixture (50 μL) contained 1× PCR buffer, 2 mmol/L MgCl_2_, 0.2 mmol/L of each dNTP, 1 μmol/L of each primer, 1U Go Taq® Hot start polymerase (Promega), and 75 ng of DNA template. Based on the nucleotide content of the primers, the annealing temperature was predicted to be 64.5°C for Deino-f-326-350 and 65°C for Deino-r-758-785. However, when PCR amplification was performed with an annealing temperature of 65°C, all *Deinococcus-Thermus* strains DNAs were successfully amplified while no amplification was detected for strains belonging to any other phyla, with the exception of *Firmicutes*. Amplification of *Firmicutes* was predicted by in silico analysis, and can be accounted for by two primer/template mismatches. To avoid nonspecific amplification of *Firmicutes*, the PCR protocol was optimized by increasing the annealing temperature to 72°C. After an initial 2 min denaturation step at 94°C, 25 cycles were performed (94°C for 30 sec, 72°C for 1 min 45 sec), followed by a final extension step at 72°C for 5 min.

In a second set of experiments, primer efficiency and specificity for environmental DNA were examined by nested PCR. First, community DNA were used as targets for PCR amplification of the 16S rRNA genes with the universal primers fD1/S17, as described in Chanal et al. (2006). This primer set recognized 39.6, 82, and 88.5% of the 139 eligible sequences of *Deinococcus-Thermus* by allowing, respectively, 0, 1, or 2 mismatches between primers and sequences. The PCR products were then purified and used as the targets in a second PCR amplification with the primers Deino-f-326-350/Deino-r-758-785. The resulting PCR products were purified, cloned into the pCR2.1-TOPO vector (TOPO TA Cloning kit; Invitrogen, Carlsbad, CA) according to the manufacturer's instructions, and electro-transformed into *E. coli* DH5α cells. Single colonies containing inserts were randomly selected and plasmids were extracted using the QIAprep Spin Miniprep Kit (Qiagen), according to manufacturer's instructions. Plasmids were sent to GATC (Germany) for Sanger sequencing, using the M13F sequencing primer. Sequence quality was ensured manually with the BioEdit Version 7.0.5.3 (Hall 1999).

### Phylogenetic analyses

To assign taxonomy, the SILVA 111 reference sequence database was downloaded and used to search each clone sequence using a Needleman–Wunsch algorithm, applying a 80% similarity cutoff to retrieve the 20 most similar sequences. Five clone sequences had no close relative in the SILVA database. These five sequences were then submitted to a Blastn query (excluding environmental sequences) using the NCBI nr database. First, we looked for hits with ≥99% similarity, and we calculated a consensus taxonomy. In the event that no hit was found with ≥99% similarity, the threshold was successively lowered in a step-wise fashion to determine at what level a taxonomy could be assigned. This process was repeated until an 80% threshold was reached. A consensus taxonomy corresponded, for example, to a defined genus if all selected hits shared the same genus.

For each of the 142 clone sequence, the two most similar sequences from the SILVA 111 reference database were selected (but with filtering to include at least one cultured bacterial sequence) to create a file of clone sequences and reference sequences (194 sequences in total). SeaView (Gouy et al. 2010) was used to align these sequences, using the included Muscle program. An initial tree was built from conserved domains, and sequences were reordered as they occurred in this tree using SeaView's tools. Alignments were checked and manually modified when necessary. This process was repeated until no problem was detected. Trees were built using Neighbor-Joining (with distances corrected using the Kimura 2-parameter method), as implemented in SeaView with 1000 bootstrap replications. Trees were plotted with TreeDyn (Chevenet et al. 2006). To determine OTUs (operational taxonomic units), sequences were pair-wise aligned by a Needleman–Wunsch algorithm, a distance matrix was computed and sequences were clustered by average linkage from 85% to 100% similarity. We used clustering with a similarity of 97% in this study.

## Results and Discussion

### In silico analysis of primer pair specificity and coverage

The Deino-f-326-350/Deino-r-758-785 primers exhibited high coverage and specificity for the phylum *Deinococcus-Thermus* (Table 2). By allowing 0, 1, or 2 mismatches between primers and sequences, the primer set, respectively, recognized 89, 95.7, and 97.2% of the 1048 sequences of *Deinococcus-Thermus*. The coverage values were high for all genera within the phylum except for *Marinithermus*.

**Table 2:** In silico analysis of the coverage obtained by the set of primers

	Total number of sequences	0 mismatch (%)	1 mismatch (%)	2 mismatches (%)
*Deinococcus-Thermus*	1048	89	95.7	97.2
*Marinithermus*	2	0	0	100
*Meiothermus*	201	88	95	97
*Oceanithermus*	15	100	100	100
*Thermus*	318	91	97	97
*Vulcanithermus*	3	67	67	100
*Deinococcus*	365	92	98	99
*Truepera*	132	80	89	92
*Proteobacteria*	222,804	<0.1	<0.1	0.3
*Firmicutes*	191,278	0	<0.1	25
*Bacteroidetes*	79,438	0	0	0.1
*Actinobacteria*	46,948	0	0	0.1
*Acidobacteria*	13,074	0	0	0.2
*Cyanobacteria*	12,752	0	0	0.1
*Chloroflexi*	10,437	0	0.9	2.4
*Tenericutes*	4234	0	0	7.8
*Nitrospirae*	2558	0	<0.1	1.6
*Fusobacteria*	2345	0	0	0.2
*Deferribacteres*	1671	0	0	1.6
*Fibrobacteres*	1116	0	0	0.1
*Chlorobi*	1101	0	0	3.3
Candidate division OD1	998	0	0	0.2
*Aquificae*	890	0.2	0.2	0.2
*Thermotogae*	770	0	0	0.1
Candidate division TM7	758	0	27.8	39.2
*Armatimonadetes*	660	0	0	1.4
BD1–5	396	0	7.6	69.9
TM6	373	0	0	4.6
TA06	310	0	0	0.3
Candidate division BRC1	265	0	0	0.8
RF3	231	0	0	4.3
Candidate division WS6	158	0	0	22.2
Candidate division KB1	78	0	0	12.8
*Spirochetes*	57	0	0	11.4
WCHB1–60	51	0	0	84.3
MVP-21	24	0	0	37.5
GAL08	17	0	0	5.9
Kazan-3B-28	13	0	0	15.4

The results for *Deinococcus-Thermus* are detailed according to the genera. The total number of sequences available in the database is indicated for each phylum and genera. The given coverage values correspond to 0, 1, or 2 mismatches between the primers and the 16S rRNA gene sequences.

Among the 731,338 sequences in the SILVA database, three (out of 222,804) *Proteobacteria* sequences and two (out of 890) *Aquificae* sequences matched exactly with the primers. Allowing one mismatch between primers and sequences increased the number of matches, to 211 sequences from Candidate division TM7 (27.8% coverage value), 89 sequences from *Chloroflexi* (0.9% coverage value), 30 sequences from BD1–5 (7.6% coverage value), and 10 sequences from *Proteobacteria*.

The coverage values increased for several phyla, when two mismatches were tolerated between primers and sequences. However, for member-rich phyla (e.g., *Proteobacteria*), the coverage values remained low (0.1–2.4%) except for *Firmicutes* (25%). Elevated coverage values were obtained for a number of small phyla, including Candidate Divisions TM7 (39.2%), WS6 (22.2%), and KB1 (12.8%); BD1–5 (69.9%), WCHB1–60 (84.3%), MVP-21 (37.5%), and Kazan-3B-28 (15.4).

### In vitro validation of primers with reference strains

Tests for primer specificity and optimization of PCR amplification conditions were performed on a set of 36 bacterial strains comprising representatives from the bacterial phyla *Actinobacteria*, *Bacteroidetes*, *Deinococcus*-*Thermus*, *Firmicutes,* and *Proteobacteria* (Table 1). When PCR amplification was performed with an annealing temperature of 72°C, *Deinococcus* and *Thermus* DNAs were successfully amplified, resulting in an expected amplicon size of 460 bp. No amplification was detected for strains belonging to any other phyla (Fig. S1).

### Contribution of the Deino-f-326-350 and Deino-r-758-785 primers for the efficient detection of *Deinococcus-Thermus* in environmental DNA

To validate the use of Deino-f-326-350 and Deino-r-758-785 primers for environmental ecology applications, primer specificity was examined using six environmental community DNAs extracted from two distinct environments: the Tataouine desert sand and Tunisian geothermal springs. A direct PCR using the Deino-f-326-350 and Deino-r-758-785 primers with a 72°C annealing temperature did not yield any product with our six environmental DNA samples. This could be related to the high annealing temperature used here, in combination with low amount of *Deinococcus-Thermus* derived DNA and large quantity of nonspecific DNA in template DNA sample. Therefore, we performed a nested PCR protocol: the universal bacterial primers fD1 and S17 were used in a first round, and the resulting amplicons were targeted in the second round with the Deino-f-326-350 and Deino-r-758-785 primers (with an annealing temperature of 72°C). This protocol resulted in PCR products of the expected size for the six DNA samples tested.

**Figure 2:**
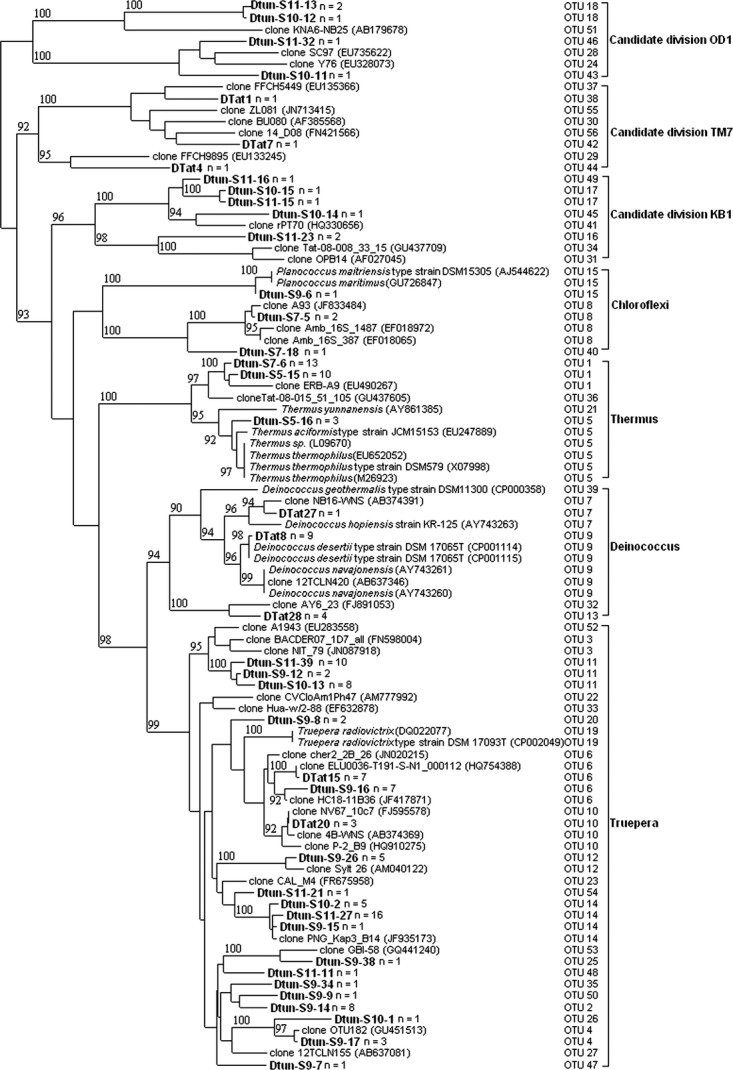
Neighbor-Joining tree based on 16S rRNA sequences recovered from Tataouine (labelled with the prefix DTat) and from hot springs 5, 7, 9, 10 and 11 (labelled with the prefixes Dtun-S5, Dtun-S7, Dtun-S9, Dtun-S10 and Dtun-S11, respectively). The tree is simplified to include one sequence per OTU and per collection site, as well as the most similar sequences from public databases. The number of sequences within each OTU is indicated at the leaves (n = x). A complete tree is shown in Figure S2.

Then, to check primer specificity, six gene libraries were constructed with the PCR products and 142 sequences were used for a phylogenetic analysis (Fig. S2); a simplified tree is shown in Figure 2. The sequences clustered into 33 OTUs at 97% sequence identity. The rarefaction curves plotted at this level did not reach an asymptote indicating that new OTUs would have appeared when increasing the number of clones sequenced (Fig. S3). One hundred twenty-four out of 142 sequences were affiliated with the targeted phylum *Deinococcus-Thermus*, whereas 18 sequences were affiliated with nontargeted phyla such as *Chloroflexi, Firmicutes,* and the Candidate Divisions KB1, TM7, and OD1. Among these 18 sequences, 15 displayed low similarity values (<93%) to known 16S rRNA gene sequences and three were related to *Firmicutes*; the latter suggests either amplification linked to primer mismatches (that could not be avoided even with an annealing temperature of 72°C) or PCR-induced artifacts. In spite of this, these nonspecific reactions occurred with a low frequency (13%) and did not prevent the detection of the targeted sequences within complex environmental DNA samples.

### Molecular diversity of taxa affiliated with *Deinococcus-Thermus* in the desert of Tataouine

All twenty-four 16S rRNA gene clone sequences from the desert of Tataouine were identified as belonging to *Deinococcales*, and could be divided into two groups: the first group affiliates with the genus *Deinococcus* and comprises 14 sequences clustered into three OTUs; the second group is affiliated with the genus *Truepera* and comprises 10 sequences clustered into two OTUs (Fig. 2).

Nine sequences from *Deinococcales* were grouped into OTU 9. Within this OTU, four sequences were closely related to *Deinococcus desertii* (with similarity values ranging from 98.3–98.7%), a bacterium previously isolated from the Tatatouine desert sand (Chanal et al. 2006). By contrast, the other sequences were affiliated to *Deinococcus hopiensis* and *D. navajonensis* (95.5–99.8% similarity), two radioresistant strains recovered from arid soils in the Sonoran hot desert (Rainey et al. 2005). Four sequences grouped into OTU 13 were not closely related to any cultured strains, and their closest neighbor (94.1–95.2% similarity) was a 16S rRNA gene sequence detected in a quartz hypolith from the Acatama desert (Lacap et al. 2011).

Ten sequences, clustered into OTUs 6 and 10, affiliated with *Truepera,* and exhibited 93–94% 16S rRNA gene sequence similarity with *Truepera radiovictrix*, the sole type strain of this genus, isolated from hot spring runoffs (Albuquerque et al. 2005). These sequences displayed higher similarity (98.4–100%) to uncultured bacteria sequences from diverse biotopes and notably from extreme environments, including: rock samples from the Black Canyon of the Chihuahuan desert (NM; Northup et al. 2010); the Mars desert research station (UT; Direito et al. 2011); sunlight-exposed biofilms from Chernobyl (Ukraine; Ragon et al. 2011); and saline biological desert crusts (China; Li et al. 2013).

Thus, application of the Deino-f-326-350 and Deino-r-758-785 primers revealed sequences that were detected in our previous study (such as *Deinococcus desertii*), as well as several new sequences that have not previously been recovered. In particular, the presence of *Truepera* in this environment was unsuspected until now.

### Molecular diversity of taxa affiliated with *Deinococcus-Thermus* in Tunisian geothermal springs

One hundred sequences belonging to the *Deinococcus-Thermus* group were recovered from hot springs 5, 7, 9, 10, and 11. These were marked by an uneven distribution among the five springs, as the 26 sequences derived from springs 5 and 7 all affiliated with *Thermales* while the 74 sequences derived from springs 9, 10, and 11 all affiliated with *Deinococcales* (Fig. 2). This result differs to some extent from that previously reported by Sayeh et al. (2010), in which *Thermales* sequences were identified in springs 5, 9, and 11, and no sequences were found to affiliate with *Deinococcales*. This discrepancy between the two studies could be explained by the fact that, for this study, new DNA extractions were performed from samples stored at −80°C, implicating that the DNA extracts were not exactly the same in both cases. The discrepancies could also be due to differences in DNA extraction procedures and PCR conditions (e.g., primer pair and/or amplification programs).

The 74 *Deinococcales* sequences recovered from springs 9, 10, and 11 all affiliated with the genus *Truepera*. Springs 10 and 11 displayed a very similar molecular diversity, whereas spring 9 had a distinct profile. Springs 10 and 11 were dominated by OTUs 11 and 14, representing, respectively, 93 and 96% of the sequences; these OTUs were also characterized by a lower than 90% similarity to cultured species. Sequences recovered from spring 9 also clustered into these two OTUs, but with much less abundance (three sequences). Sequences from OTU 14 were most closely related (95–99.8%) to sequences recovered from “hot” environments such as alkaline hot springs (JF935173, Papua New Guinea), arid soils of northwestern China (FR849462), and hot Calamita ferromagnetic sand (Perfumo et al. 2011). Sequences from OTU 11 were most closely related (95–96% similarity) to uncultured bacteria derived from an impressive number of saline and hypersaline environments, including: French Guiana coast mud (KC010001); the northern subtropical Pacific Ocean (Eiler et al. 2011); hypersaline microbial mats (Mexico, JN501803); north Pacific subtropical gyres (Pham et al. 2008); activated sludge from a seawater-processing wastewater treatment plant (Sánchez et al. 2011); the northeast subarctic Pacific Ocean (HQ674210); coastal sediments of the Ariake Sea (Japan, AB560052); the Xiao Chaidan salt lake (China, HM128252); tailing material from Chanaral Bay (Acatama desert, HF558617); hypersaline sediments from Lake Kasin (Russia; Emmerich et al. 2012); marine sponge (Florida; Montalvo and Hill 2011); and the Sapelo Island salt marsh (GA, AY711411). Despite their great geographic separation, springs 9 and 10 (located near Tozeur, at the border of the “Chott el-Djerid” endorheic salt lake) and 11 (located near Korbous, on the Mediterranean Sea) each showed elevated salinity (3, 5, and 11 g/L, respectively). Taken together, these data strongly suggest that sequences from OTUs 11 and 14 belong to ubiquitous halophilic *Truepera* found in saline environments worldwide.

Nine OTUs related to *Truepera* (2, 4, 6, 12, 20, 25, 35, 47, and 50), and which represent 30 sequences, were detected exclusively in spring 9. Sequences belonging to the OTUs 2, 4, 12, 25, 47, and 50 were most closely related to uncultured organisms derived from marine environments, such as surfaces of marine macroalgae (GU451513; Lachnit et al. 2011) and marine sandy sediments from the North Sea (AM040122; Musat et al. 2006). Bacteria belonging to these OTUs may correspond to salt-tolerant species. This hypothesis is consistent with the location of spring 9 near the endorheic salt lake “Chott el-Djerid”. The OTU 6 encompassed six sequences that were also detected in spring 9, and which displayed close relationships with sequences detected in several extreme environments such as deserts and radioactive sites (see above). This OTU was singular in that it was additionally represented in the Tataouine desert sample; this phenomenon could be the signature of exchanges between the Tataouine site and spring 9, possibly occurring through the dust and sand storms that frequently occur in this region.

The 26 *Thermales* sequences derived from spring 5 (13 sequences) and spring 7 (13 sequences) all affiliated with the genus *Thermus* and clustered into two OTUs. OTU 1 was the most abundant and accounted for 77% of the sequences from spring 5, and 100% of the sequences from spring 7. Sequences from this OTU have no close cultured neighbor, and exhibit high similarity (96.1–97.0%) with a sequence detected in hot mineral soils (Antarctica; Soo et al. 2009). In addition, three sequences from spring 5 clustered into OTU 5 and were closely related to *Thermus thermophilus* (97.4–99.6% similarity) and *Thermus arciformis* (97.6% similarity), two thermophilic strains isolated from hot springs (Murzina et al. 1988; Zhang et al. 2010). As most *Deinococcus* species are mesophilic or moderately thermophilic, the absence of these bacteria from springs 5 and 7 could be explained by the elevated temperatures recorded at these sites (73°C and 70°C, respectively).

## Conclusions

We have demonstrated that the primers developed for this study are highly specific and allow the detection of *Deinococcus-Thermus* sequences within environmental samples. They represent a powerful tool to detect novel *Deinococcus*-*Thermus* sequences through the sequencing of a limited number of clones, which will provide new insight into *Deinococcus*-*Thermus* molecular diversity in extreme environments. In line with this, our results indicate the presence of previously undescribed salt-tolerant bacteria in three springs.

These primers could be used in ecological studies for a rapid screening of environmental DNA samples, and could also be applicable to clarify the biogeography of the *Deinococcus*-*Thermus* phylum.

## Supporting Information

**Figure S1.** PCR amplification using Deino-f-326-350 and Deino-r-758-785 primers and genomic DNA of diverse *Deinococcus, Meiothermus, Thermus, Paenibacillus, Bacillus and Lysinibacillus* strains.

**Figure S2**. Neighbor-Joining tree (1000 replicates) based on 16S rRNA gene sequences recovered from Tataouine (labelled with the prefix DTat) and from hot springs 5, 7, 9, 10 and 11 (labelled with the prefixes Dtun-S5, Dtun-S7, Dtun-S9, Dtun-S10 and Dtun-S11, respectively). The tree includes the most similar sequences from public databases.

**Figure S3.** Rarefaction curves of the OTUs at 97% sequence similarity.

## References

[b1] Albuquerque L, Simões C, Nobre MF, Pino NM, Battista JR, Silva MT (2005). *Truepera radiovictrix* gen. nov., sp. nov., a new radiation resistant species and the proposal of *Trueperaceae* fam. nov. FEMS Microbiol. Lett.

[b2] Asker D, Awad TS, McLandsborough L, Beppu T, Ueda K (2011). *Deinococcus depolymerans* sp. nov., a gamma- and UV-radiation-resistant bacterium, isolated from a naturally radioactive site. Int. J. Syst. Evol. Microbiol.

[b3] Auchtung TA, Shyndriayeva G, Cavanaugh CM (2011). 16S rRNA phylogenetic analysis and quantification of *Korarchaeota* indigenous to the hot springs of Kamchatka, Russia. Extremophiles.

[b4] Bjornsdottir SH, Petursdottir SK, Hreggvidsson GO, Skirnisdottir S, Hjorleifsdottir S, Arnfinnsson J (2009). *Thermus islandicus* sp. nov., a mixotrophic sulfur-oxidizing bacterium isolated from the Torfajokull geothermal area. Int. J. Syst. Evol. Microbiol.

[b5] Brock TD, Freeze H (1969). *Thermus aquaticus* gen. n. and sp. n., a nonsporulating extreme thermophile. J. Bacteriol.

[b55] Brooks BW, Murray RGE (1981). Nomenclature for *Micrococcus radiodurans* and other radiation resistant cocci: *Deinococcaceae* fam. nov. and *Deinococcus* gen. nov., including five species. Int. J. Syst. Bacteriol.

[b6] Callegan RP, Nobre MF, McTernan PM, Battista JR, Navarro-González R, McKay CP (2008). Description of four novel psychrophilic, ionizing radiation-sensitive *Deinococcus* species from alpine environments. Int. J. Syst. Evol. Microbiol.

[b7] Chanal A, Chapon V, Benzerara K, Barakat M, Christen R, Achouak W (2006). The desert of Tataouine: an extreme environment that hosts a wide diversity of microorganisms and radiotolerant bacteria. Environ. Microbiol.

[b63] Chapon V, Piette L, Vesvres MH, Coppin F, Christen C, Le Marrec R (2012). Microbial diversity in contaminated soils along the T22 trench of the Chernobyl experimental platform. Appl. Geochem.

[b8] Chaturvedi R, Archana G (2012). Novel 16S rRNA based PCR method targeting *Deinococcus* spp. and its application to assess the diversity of deinococcal populations in environmental samples. J. Microbiol. Methods.

[b9] Chevenet F, Brun C, Banuls AL, Jacq B, Christen R (2006). TreeDyn: towards dynamic graphics and annotations for analyses of trees. BMC Bioinform.

[b10] da Costa MS, Rainey FA, Nobre MF, Dworkin M, Falkow S, Rosenberg E, Schleifer KH, Stackebrandt E (2006). The genus *Thermus* and relatives. The prokaryotes. A handbook on the biology of bacteria.

[b11] Cox MM, Battista JR (2005). *Deinococcus radiodurans* – the consummate survivor. Nat. Rev. Microbiol.

[b12] Direito S, Ehrenfreund P, Marees A, Staats M, Foing B, Röling W (2011). A wide variety of putative extremophiles and large beta-diversity at the Mars Desert Research Station (Utah). Int. J. Astrobiol.

[b13] Duodu S, Larsson P, Sjödin A, Forsman M, Colquhoun DJ (2012). The distribution of *Francisella*-like bacteria associated with coastal waters in Norway. Microb. Ecol.

[b14] Eiler A, Hayakawa DH, Rappé MS (2011). Non-random assembly of bacterioplankton communities in the subtropical north pacific ocean. Front. Microbiol.

[b15] Ekman JV, Raulio M, Busse HJ, Fewer DP, Salkinoja-Salonen M (2011). *Deinobacterium chartae* gen. nov., sp. nov., an extremely radiation-resistant, biofilm-forming bacterium isolated from a Finnish paper mill. Int. J. Syst. Evol. Microbiol.

[b16] Emmerich M, Bhansali A, Lösekann-Behrens T, Schröder C, Kappler A, Behrens S (2012). Abundance, distribution, and activity of Fe(II)-oxidizing and Fe(III)-reducing microorganisms in hypersaline sediments of Lake Kasin, southern Russia. Appl. Environ. Microbiol.

[b54] Ferreira AC, Nobre MF, Rainey FA, Silva MT, Wait R, Burghardt J (1997). *Deinococcus geothermalis* sp. nov. and *Deinococcus murrayi* sp. nov., two extremely radiation-resistant and slightly thermophilic species from hot springs. Int. J. Syst. Bacteriol.

[b17] Fredrickson JK, Zachara JM, Balkwill DL, Kennedy D, Li SM, Kostandarithes HM (2004). Geomicrobiology of high-level nuclear waste-contaminated vadose sediments at the Hanford site, Washington state. Appl. Environ. Microbiol.

[b18] Gans JD, Dunbar J, Eichorst SA, Gallegos-Graves LV, Wolinsky M, Kuske CR (2012). A robust PCR primer design platform applied to the detection of Acidobacteria Group 1 in soil. Nucleic Acids Res.

[b19] Gouy M, Guindon S, Gascuel O (2010). SeaView version 4: a multiplatform graphical user interface for sequence alignment and phylogenetic tree building. Mol. Biol. Evol.

[b20] de Groot A, Chapon V, Servant P, Christen R, Saux MF, Sommer S (2005). *Deinococcus deserti* sp. nov., a gamma-radiation-tolerant bacterium isolated from the Sahara Desert. Int. J. Syst. Evol. Microbiol.

[b21] Hall TA (1999). BioEdit: a user-friendly biological sequence alignment editor and analysis program for Windows 95/98/NT. Nucleic Acids Symp. Ser.

[b22] Hirsch P, Gallikowski CA, Siebert J, Peissl K, Kroppenstedt R, Schumann P (2004). *Deinococcus frigens* sp. nov., *Deinococcus saxicola* sp. nov., and *Deinococcus marmoris* sp. nov., low temperature and draught-tolerating, UV-resistant bacteria from continental Antarctica. Syst. Appl. Microbiol.

[b23] Im WT, Jung HM, Ten LN, Kim MK, Bora N, Goodfellow M (2008). *Deinococcus aquaticus* sp. nov., isolated from fresh water, and *Deinococcus caeni* sp. nov., isolated from activated sludge. Int. J. Syst. Evol. Microbiol.

[b24] Janssen PH (2006). Identifying the dominant soil bacterial taxa in libraries of 16S rRNA and 16S rRNA genes. Appl. Environ. Microbiol.

[b25] Kämpfer P, Lodders N, Huber B, Falsen E, Busse HJ (2008). *Deinococcus aquatilis* sp. nov., isolated from water. Int. J. Syst. Evol. Microbiol.

[b57] Kobatake M, Tanabe S, Hasegawa S (1973). New Micrococcus radioresistant red pigment, isolated from Lama glama feces, and its use as microbiological indicator of radiosterilization. C. R. Seances Soc. Biol. Fil.

[b26] Lacap DC, Warren-Rhodes KA, McKay CP, Pointing SB (2011). Cyanobacteria and chloroflexi-dominated hypolithic colonization of quartz at the hyper-arid core of the Atacama Desert, Chile. Extremophiles.

[b27] Lachnit T, Meske D, Wahl M, Harder T, Schmitz R (2011). Epibacterial community patterns on marine macroalgae are host-specific but temporally variable. Environ. Microbiol.

[b28] Lagier JC, Armougom F, Million M, Hugon P, Pagnier I, Robert C (2012). Microbial culturomics: paradigm shift in the human gut microbiome study. Clin. Microbiol. Infect.

[b29] Lee SH, Cho JC (2011). Group-specific PCR primers for the phylum Acidobacteria designed based on the comparative analysis of 16S rRNA gene sequences. J. Microbiol. Methods.

[b58] Lewis NF (1973). Radio-resistant *Micrococcus radiophilus* sp. nov. isolated from irradiated Bombay duck (*Harpodon nehereus*. Curr. Sci. (Bangalore).

[b30] Li K, Liu R, Zhang H, Yun J (2013). The diversity and abundance of bacteria and oxygenic phototrophs in saline biological desert crusts in Xinjiang, Northwest China. Microb. Ecol.

[b59] Loginova LG, Egorova LA, Golovacheva RS, Seregina LM (1984). *Thermus ruber* sp. nov., nom. rev. Int. J. Syst. Bacteriol.

[b60] Manaia CM, Hoste B, Gutiérrez MC, Gillis M, Ventosa A, Kersters K, da Costa MS (1994). Halotolerant *Thermus* strains from marine and terrestrial hot springs belong to *Thermus thermophilus* (ex Oshima and Imahori, 1974) nom. rev. emend. Syst. Appl. Microbiol.

[b31] Montalvo NF, Hill RT (2011). Sponge-associated bacteria are strictly maintained in two closely related but geographically distant sponge hosts. Appl. Environ. Microbiol.

[b32] Mühling M, Woolven-Allen J, Murrell JC, Joint I (2008). Improved group-specific PCR primers for denaturing gradient gel electrophoresis analysis of the genetic diversity of complex microbial communities. ISME J.

[b33] Murzina NV, Vorozheykina DP, Matvienko NI (1988). Nucleotide sequence of *Thermus thermophilus* HB8 gene coding 16S rRNA. Nucleic Acids Res.

[b34] Musat N, Werner U, Knittel K, Kolb S, Dodenhof T, van Beusekom JE (2006). Microbial community structure of sandy intertidal sediments in the North Sea, Sylt-Rømø Basin, Wadden Sea. Syst. Appl. Microbiol.

[b62] Nobre MF, Trüper HG, da Costa MS (1996). Transfer of *Thermus ruber* (Loginova et al. 1984), *Thermus silvanus* (Tenreiro et al. 1995), and *Thermus chliarophilus* (Tenreiro et al. 1995) to *Meiothermus* gen. nov. as *Meiothermus ruber* comb. nov., *Meiothermus silvanus* comb. nov., and *Meiothermus chliarophilus* comb. nov., respectively, and emendation of the genus *Thermus*. Int. J. Syst. Bacteriol.

[b35] Northup DE, Snider JR, Spilde MN, Porter ML, Boston JL, van de Kamp PJ (2010). Diversity of rock varnish bacterial communities from Black Canyon, New Mexico. J. Geophys. Res.

[b36] Oshima T, Imahori K (1974). Description of *Thermus thermophilus* (Yoshida and Oshima) com. nov., a non-sporulating thermophilic bacterium from a Japanese thermal spa. Int. J. Syst. Bacteriol.

[b56] Oyaizu H, Stackebrandt E, Schleifer KH, Ludwig W, Pohla H, Hirata A (1987). A radiation-resistant rod-shaped bacterium *Deinobacter grandis* gen. nov., sp. nov., with peptidoglycan containing ornithine. Int. J. Syst. Bacteriol.

[b37] Perfumo A, Cockell C, Elsaesser A, Marchant R, Kminek G (2011). Microbial diversity in Calamita ferromagnetic sand. Environ. Microbiol. Rep.

[b38] Pham VD, Konstantinidis KT, Palden T, DeLong EF (2008). Phylogenetic analyses of ribosomal DNA-containing bacterioplankton genome fragments from a 4000 m vertical profile in the North Pacific Subtropical Gyre. Environ. Microbiol.

[b39] Ragon M, Restoux G, Moreira D, Moller AP, Lopez-Garcia P, Møller AP (2011). Sunlight-exposed biofilm microbial communities are naturally resistant to chernobyl ionizing-radiation levels. PLoS ONE.

[b40] Rainey FA, Nobre MF, Schumann P, Stackebrandt E, da Costa MS (1997). Phylogenetic diversity of the deinococci as determined by 16S ribosomal DNA sequence comparison. Int. J. Syst. Bacteriol.

[b41] Rainey FA, Ray K, Ferreira M, Gatz BZ, Nobre MF, Bagaley D (2005). Extensive diversity of ionizing-radiation-resistant bacteria recovered from Sonoran Desert soil and description of nine new species of the genus *Deinococcus* obtained from a single soil sample. Appl. Environ. Microbiol..

[b42] Rainey FA, Ferreira M, Nobre MF, Ray K, Bagaley D, Earl AM (2007). *Deinococcus peraridilitoris* sp. nov., isolated from a coastal desert. Int. J. Syst. Evol. Microbiol.

[b43] Sánchez O, Garrido L, Forn I, Massana R, Maldonado MI, Mas J (2011). Molecular characterization of activated sludge from a seawater-processing wastewater treatment plant. Microb. Biotechnol.

[b44] Sayeh R, Birrien JL, Alain K, Barbier G, Hamdi M, Prieur D (2010). Microbial diversity in Tunisian geothermal springs as detected by molecular and culture-based approaches. Extremophiles.

[b45] Slade D, Radman M (2011). Oxidative stress resistance in *Deinococcus radiodurans*. Microbiol. Mol. Biol. Rev.

[b46] Soo RM, Wood SA, Grzymski JJ, McDonald IR, Cary SC (2009). Microbial biodiversity of thermophilic communities in hot mineral soils of Tramway Ridge, Mount Erebus, Antarctica. Environ. Microbiol.

[b47] Stach JE, Maldonado LA, Ward AC, Goodfellow M, Bull AT (2003). New primers for the class Actinobacteria: application to marine and terrestrial environments. Environ. Microbiol.

[b61] Suresh K, Reddy GSN, Sengupta S, Shivaji S (2004). *Deinococcus indicus* sp. nov., an arsenic-resistant bacterium from an aquifer in West Bengal. India. Int. J. Syst. Evol. Microbiol.

[b48] Vajna B, Kanizsai S, Kéki Z, Márialigeti K, Schumann P, Tóth EM (2012). *Thermus composti* sp. nov., isolated from oyster mushroom compost. Int. J. Syst. Evol. Microbiol.

[b49] Widmer F, Seidler RJ, Gillevet PM, Watrud LS, Di Giovanni GD (1998). A highly selective PCR protocol for detecting 16S rRNA genes of the genus Pseudomonas (sensu stricto) in environmental samples. Appl. Environ. Microbiol.

[b50] Yoo SH, Weon HY, Kim SJ, Kim YS, Kim BY, Kwon SW (2010). *Deinococcus aerolatus* sp. nov. and *Deinococcus aerophilus* sp. nov., isolated from air samples. Int. J. Syst. Evol. Microbiol.

[b51] Yu TT, Yao JC, Ming H, Yin YR, Zhou EM, Liu MJ (2012). *Thermus tengchongensis* sp. nov., isolated from a geothermally heated soil sample in Tengchong, Yunnan, south-west China. Antonie Van Leeuwenhoek.

[b52] Yuan M, Zhang W, Dai S, Wu J, Wang Y, Tao T (2009). *Deinococcus gobiensis* sp. nov., an extremely radiation-resistant bacterium. Int. J. Syst. Evol. Microbiol.

[b53] Zhang XQ, Ying Y, Ye Y, Xu XW, Zhu XF, Wu M (2010). *Thermus arciformis* sp. nov., a thermophilic species from a geothermal area. Int. J. Syst. Evol. Microbiol.

