# Supplementary material for: Exploration of Deinococcus-Thermus molecular diversity by novel group-specific PCR primers
Source: Microbiologyopen. 2013 Aug 29;2(5):862–72. doi: 10.1002/mbo3.119 (PMC3831646; doi:10.1002/mbo3.119)
Supplement: Supplementary file 1 [file mbo30002-0862-SD1.pdf]

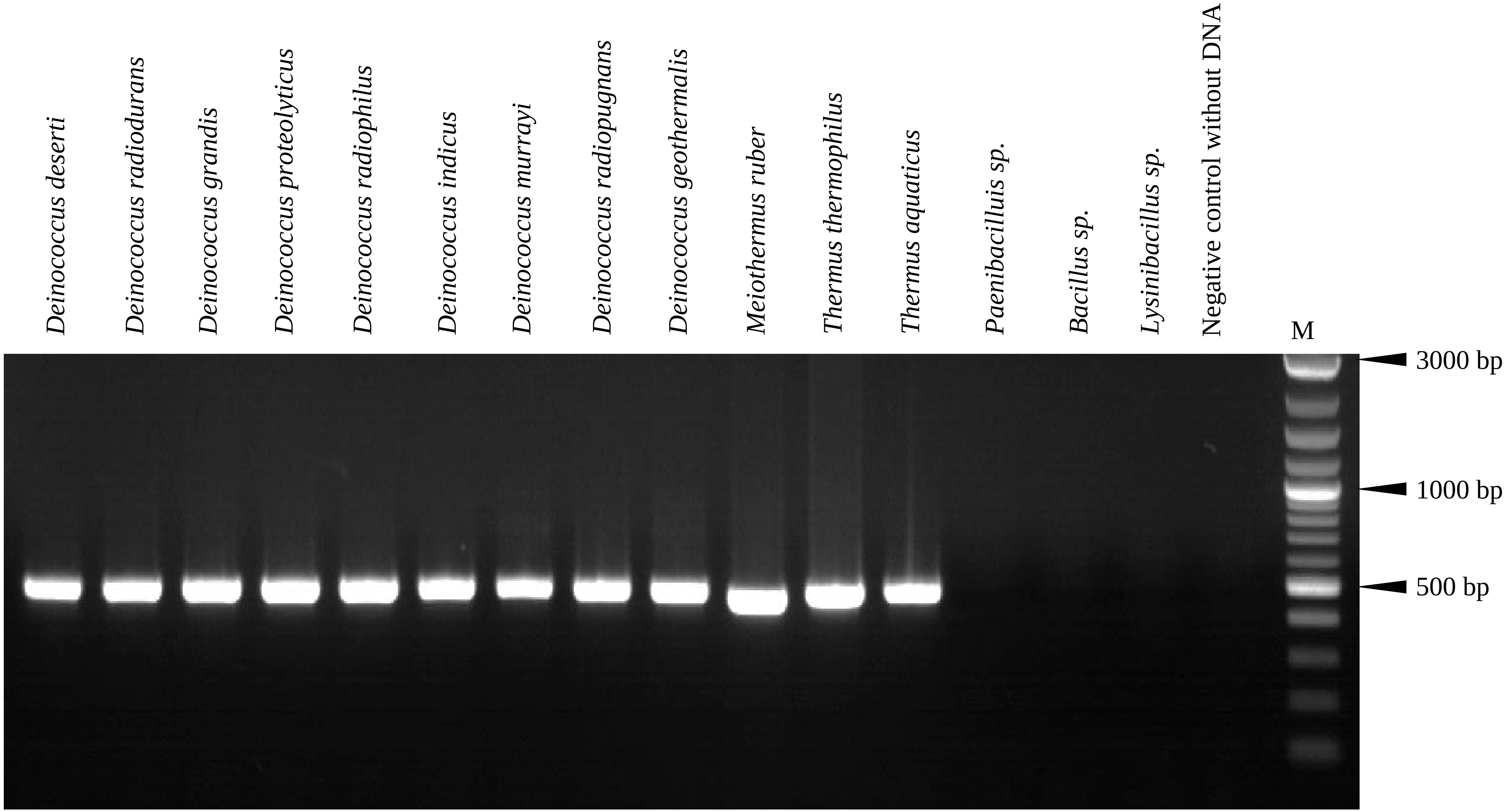

Figure S1: PCR amplification using Deino-f-326-350 and Deino-r-758-785 primers and genomic DNA of diverse *Deinococcus, Meiothermus, Thermus, Paenibacillus, Bacillus and Lysinibacillus* strains.
