# Supplementary figures and images for: Exploration of Deinococcus-Thermus molecular diversity by novel group-specific PCR primers
Source: Microbiologyopen. 2013 Aug 29;2(5):862–72. doi: 10.1002/mbo3.119 (PMC3831646; doi:10.1002/mbo3.119)

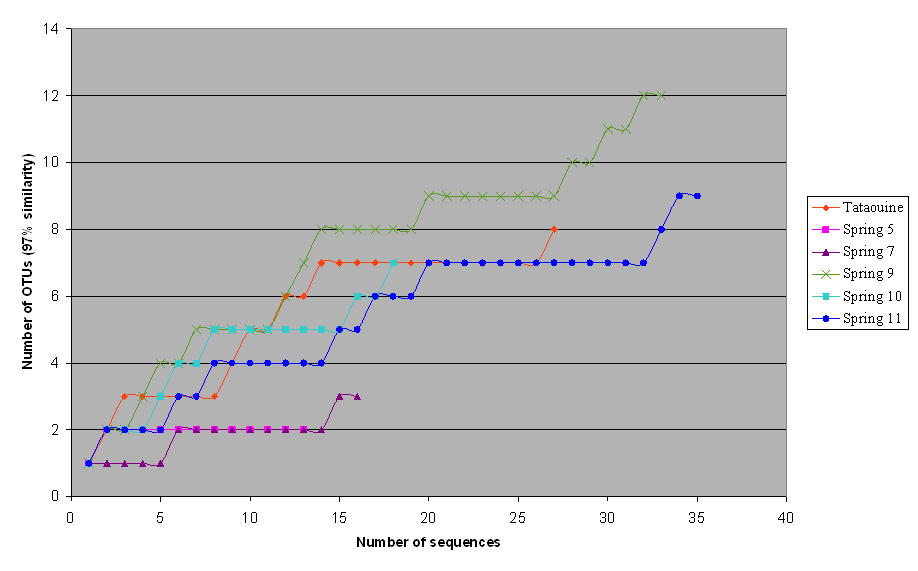

Supplement: Supplementary file 3 [file mbo30002-0862-SD3.tif]
